# Supplementary material for: Anti-Tumor Effect of Inhibition of DNA Damage Response Proteins, ATM and ATR, in Endometrial Cancer Cells
Source: Cancers (Basel). 2019 Dec 1;11(12):1913. doi: 10.3390/cancers11121913 (PMC6966633; doi:10.3390/cancers11121913)
Supplement: Supplementary file 1 [file cancers-11-01913-s001.pdf]

cancers

MDPI

Article

Anti-Tumor Effect of Inhibition of DNA Damage Response Proteins, ATM and ATR, in Endometrial Cancer Cells

Makoto Takeuchi [1], Michihiro Tanikawa [1], Kazunori Nagasaka [1,2,*], Katsutoshi Oda [1], Yoshiko Kawata [1], Shinya Oki [1], Chuwa Agapiti [1], Kenbun Sone [1], Yuko Miyagawa [2], Haruko Hiraike [2], Osamu Wada-Hiraike [1], Hiroyuki Kuramoto [3], Takuya Ayabe [2], Yutaka Osuga [1] and Tomoyuki Fujii [1]

Supplementary Figure S1. Western blot show all the bands with all the molecular weight markers.

Figure 2. DXR and CDDP activated both ATR/Chk1 and ATM/Chk2 pathways in HEC-6 endometrial cancer cells, which was canceled by each inhibitor. The medium was replaced with fresh medium containing (**a**) VE822 (0–1000 nM) or (**b**) KU60019 (0–100 μM) for 1 h before DXR (1 μM, 6 h) or CDDP (20 μM, 6 h) treatments. DXR, doxorubicin; CDDP, cisplatin. The lower histograms show the quantitative analyses of the intensities of the phosphoprotein bands from three independent experiments with SD indicated.

Figure 5. Irradiation activated both ATR/Chk1 and ATM/Chk2 pathways in HEC-6 endometrial cancer cells, which was then canceled by each inhibitor. (a) Proteins were extracted from HEC-6 cells after irradiation for a period from 15 min to 72 h. The medium was replaced by fresh medium, and the inhibitor (**b**) VE822 (0–1000 nM) or (**c**) KU60019 (0–10 μM) was added for 1 h before irradiation (10 Gy). All proteins were extracted 2 h after irradiation. The lower histograms show the quantitative analyses of the intensities of the phosphoproteins bands from three independent experiments with SD indicated.

Figure 8. Evaluation of the effect of the combination of the ATR inhibitor and the Chk1 inhibitor by immunoblotting and immunofluorescence. HEC-6 (upper figure) and HEC-1B (lower figure) were treated with VE822 (1 μM) and AZD7762 (30–60 nM) for 24 h before protein extraction.

Figure 2(a) DXR (1μM, 6h) ± VE822

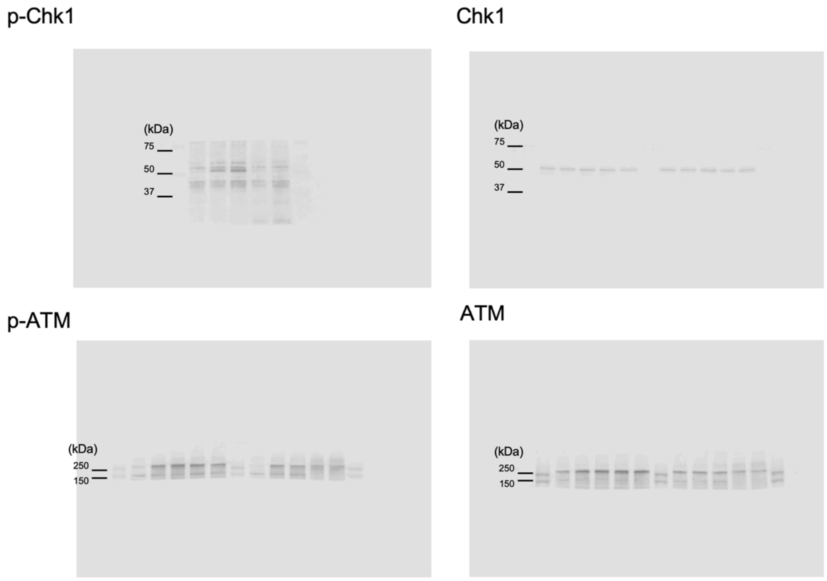

DXR+VE822 data are shown in the right lanes
pCHk1 is the repeated data gel.

Figure 2(a) DXR (1μM, 6h) ± VE822

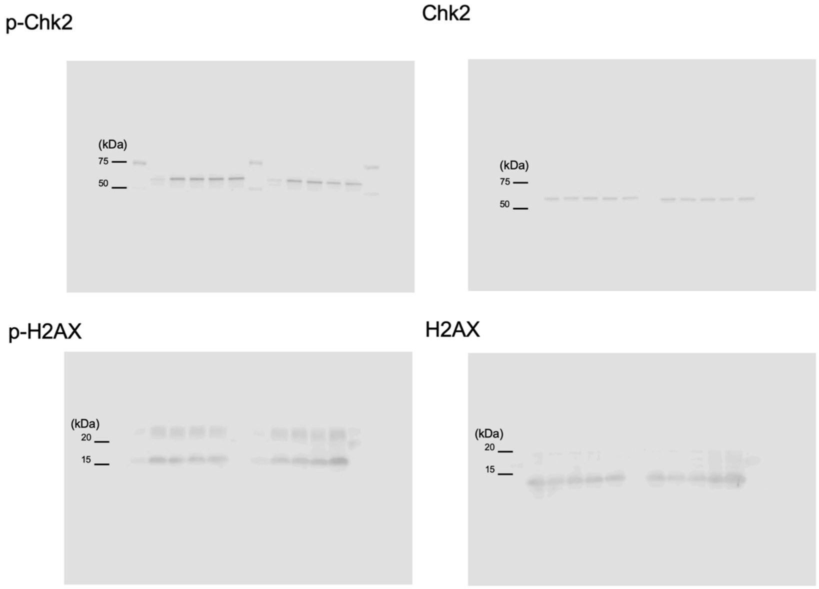

DXR+VE822 data are shown in the right lanes.

Figure 2(a) DXR (1μM, 6h) ± VE822

actin

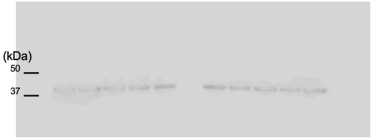

DXR+VE822 data are shown in the right lanes.

Figure 2(a) DXR (1μM, 6h) ± KU60019

p-Chk1

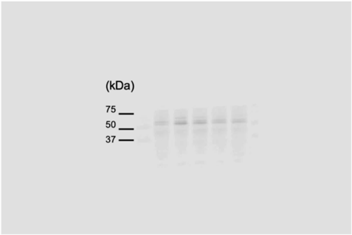

Chk1

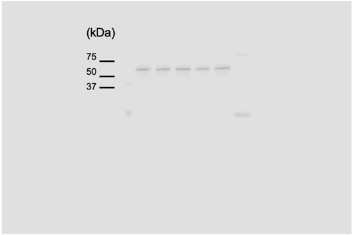

p-ATM

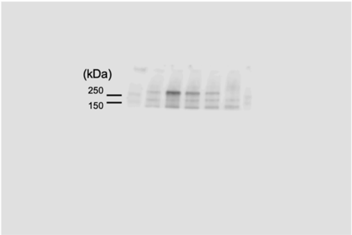

ATM

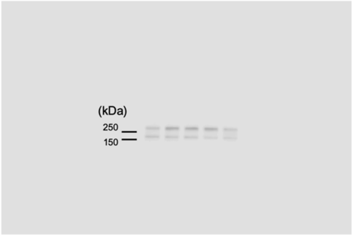

Figure 2(a) DXR (1μM, 6h) ± KU60019

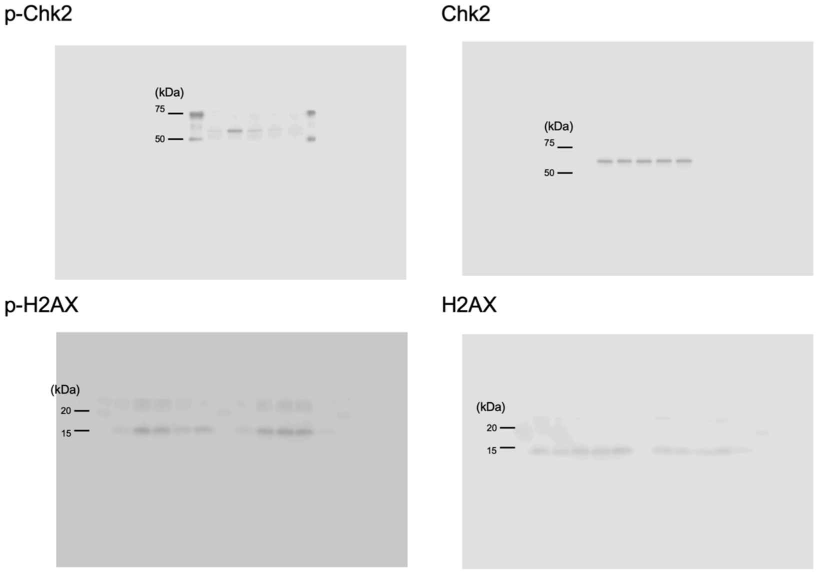

DXR+KU60019 data are shown in the left lanes.

Figure 2(a) DXR (1μM, 6h) ± KU60019

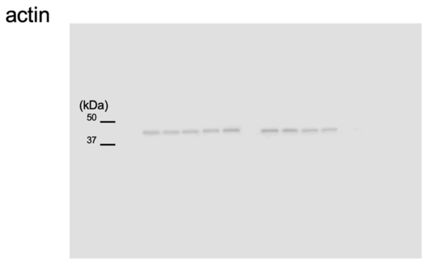

DXR+KU60019 data are shown in the left lanes.

Figure 2(b) CDDP (20μM, 6h) ± VE822

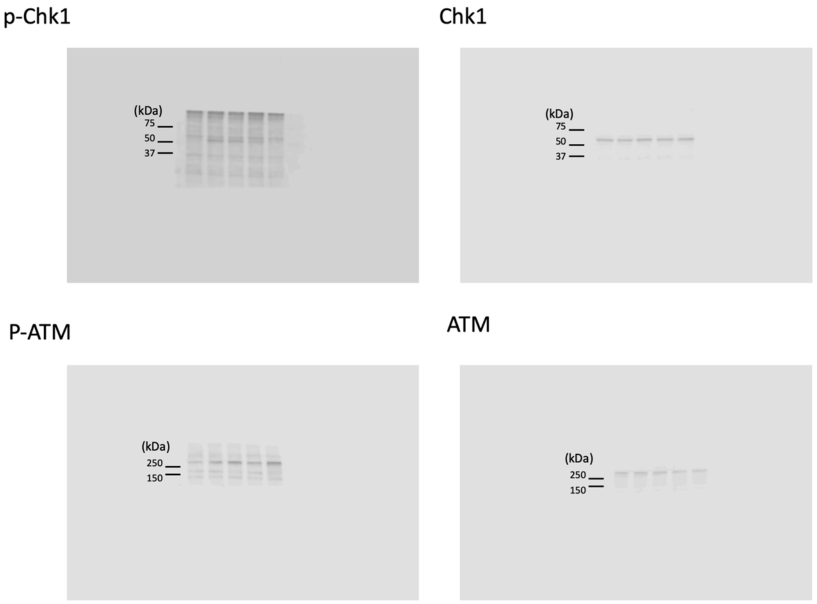

Figure 2(b) CDDP (20μM, 6h) ± VE822

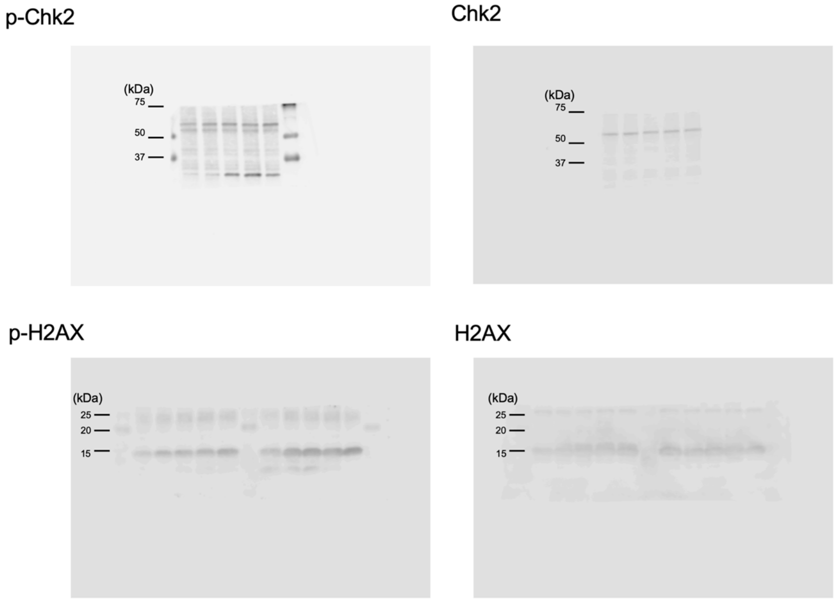

pH2AX and H2AX are repeated twice using the same samples.

Figure 2(b) CDDP (20μM, 6h) ± VE822

actin

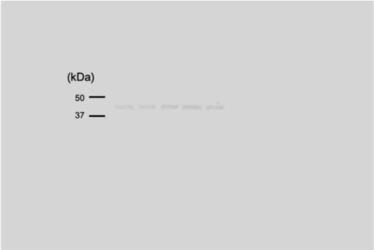

Figure 2(b) left panel

Figure 2(b) CDDP (20μM, 6h) ± KU60019

p-Chk1

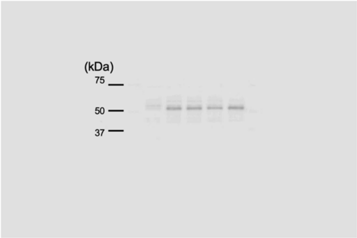

Chk1

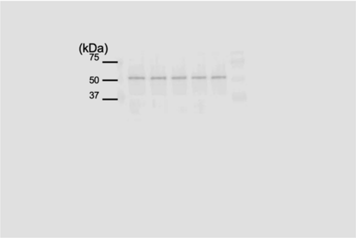

p-ATM

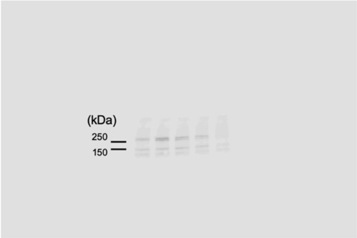

ATM

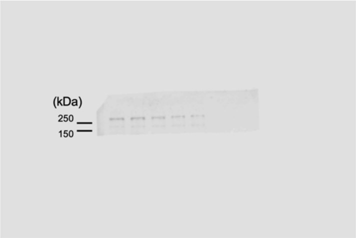

Figure 2(b) CDDP (20μM, 6h) ± KU60019

p-Chk2

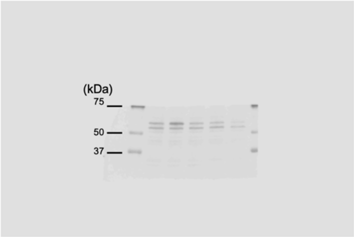

Chk2

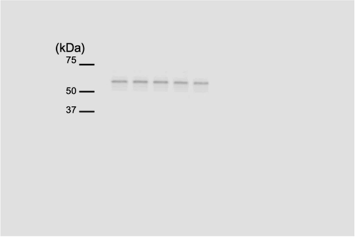

p-H2AX

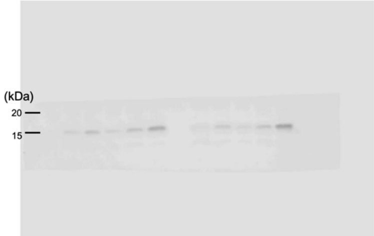

H2AX

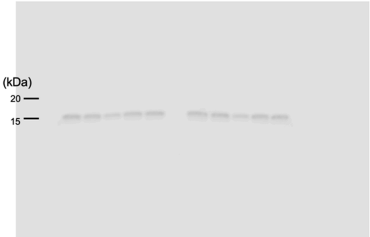

pH2AX and H2AX are repeated twice using the same samples.

Figure 2(b) CDDP (20μM, 6h) ± KU60019

actin

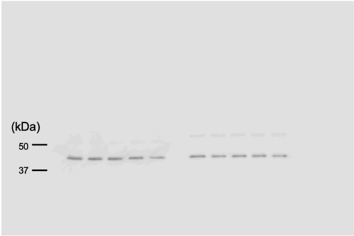

Actin is repeated twice using the same samples

Figure 5(a)

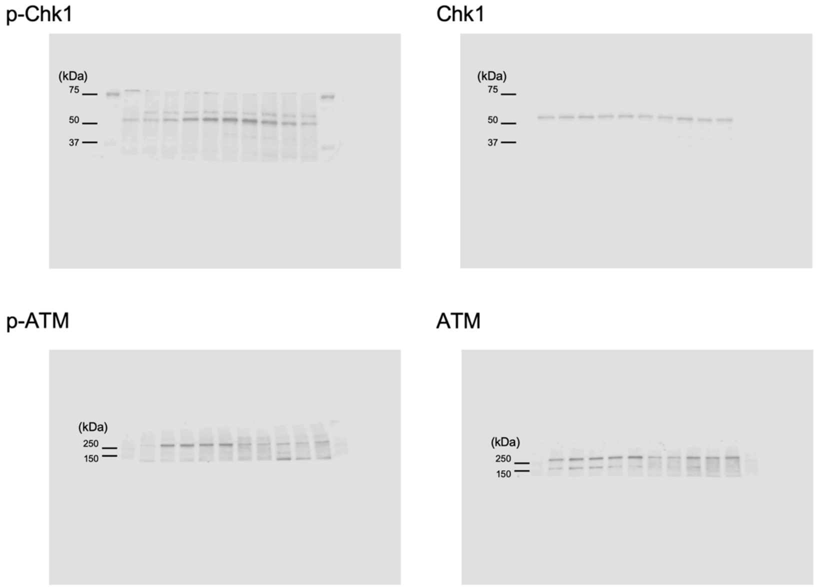

Figure 5(a)

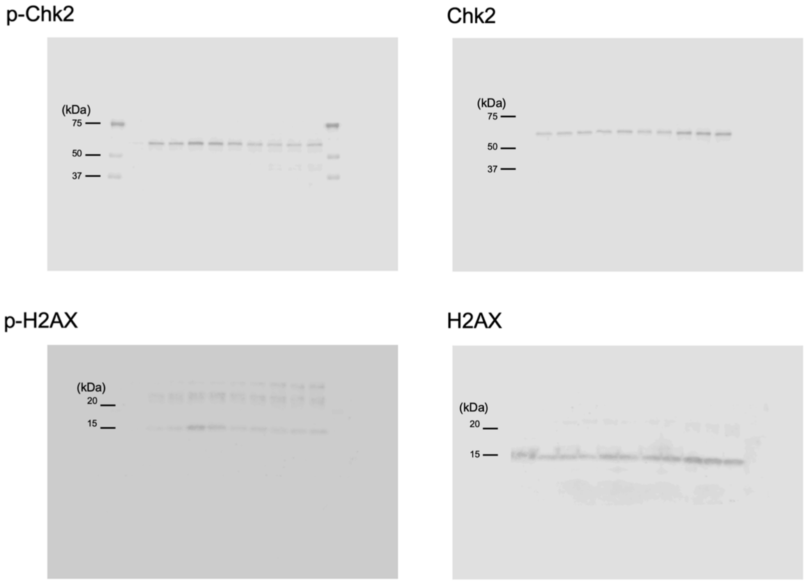

Figure 5(a)

actin

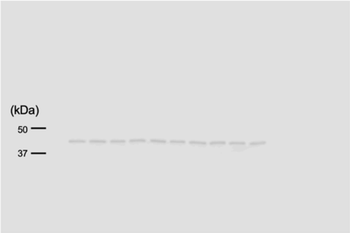

Figure 5(b)(C)

p-Chk1

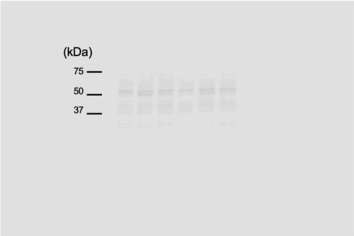

Chk1

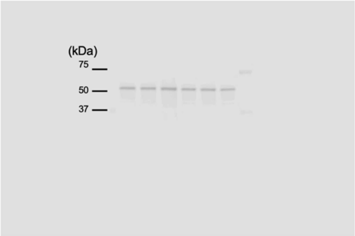

p-ATM

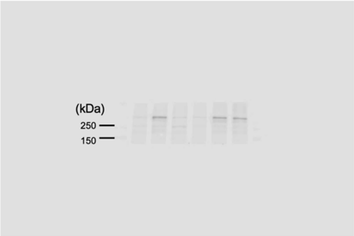

ATM

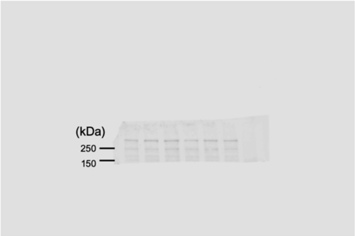

Figure(c) to (b) from left.

Figure 5(b)(C)

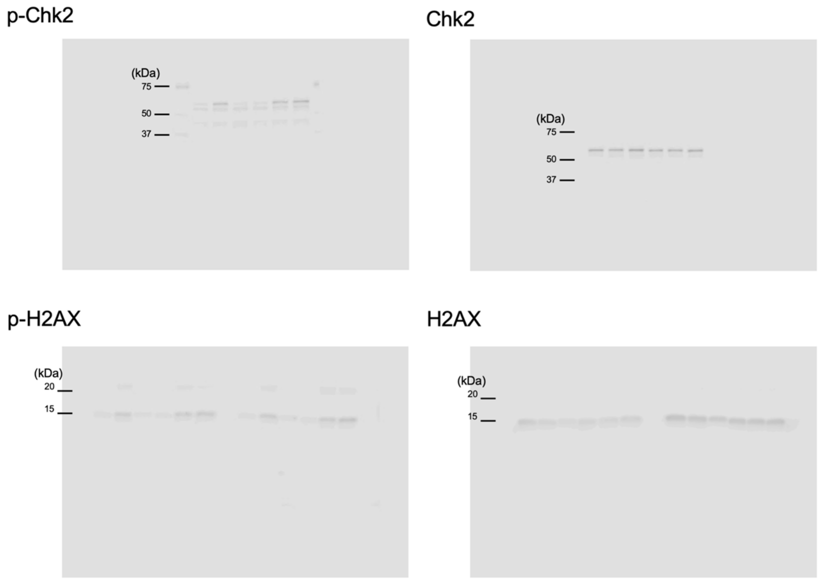

Figure(c) to (b) from left.
pH2AX and H2AX are repeated twice using the same samples.

Figure 5(b)(C)

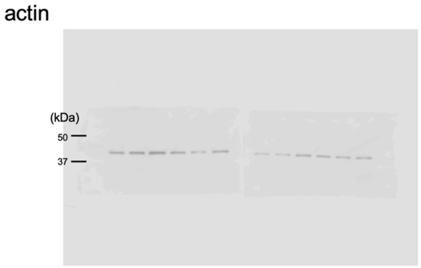

Figure(c) to (b) from left.
Actin is repeated twice using the same samples.

Figure 8

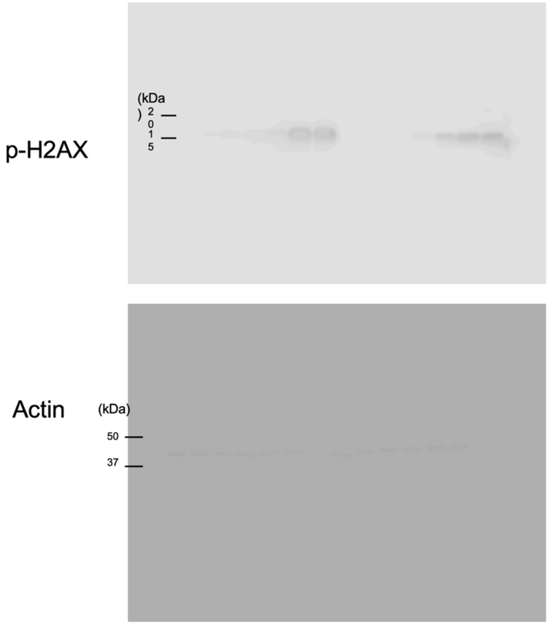

HEC-6 data are shown in the right and HEC-1B are in the left lanes.

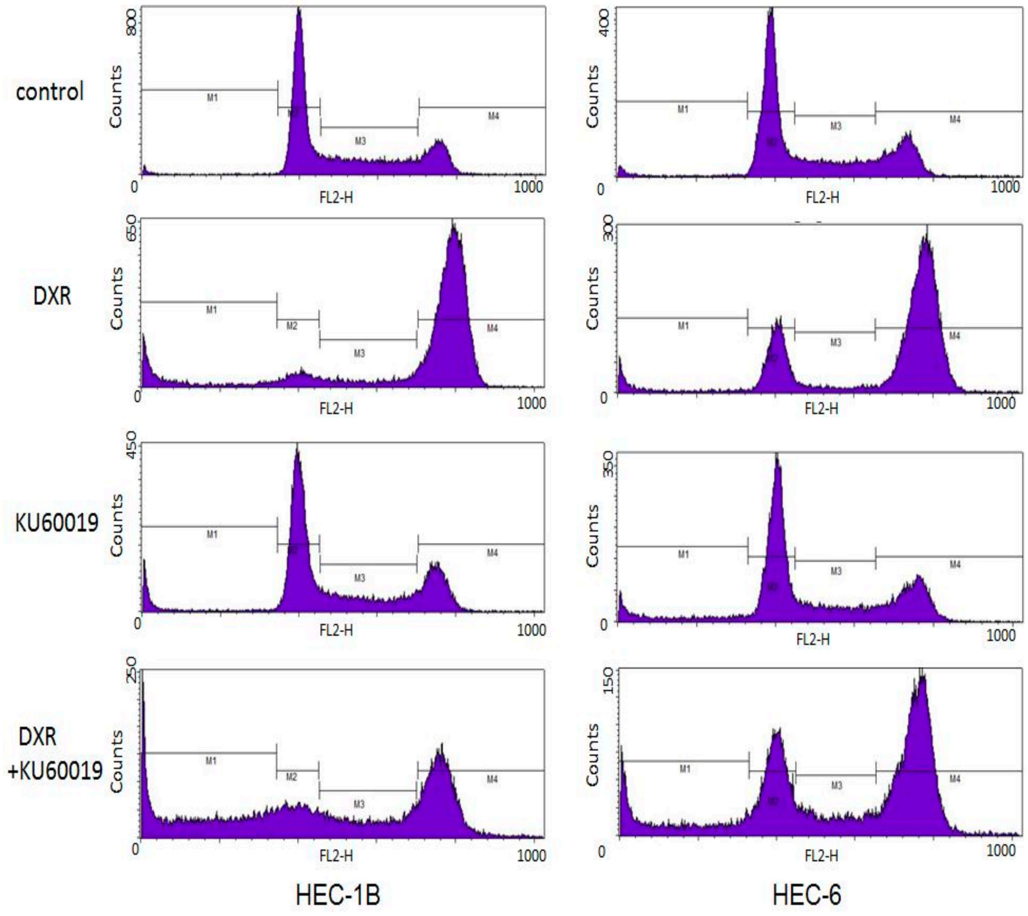

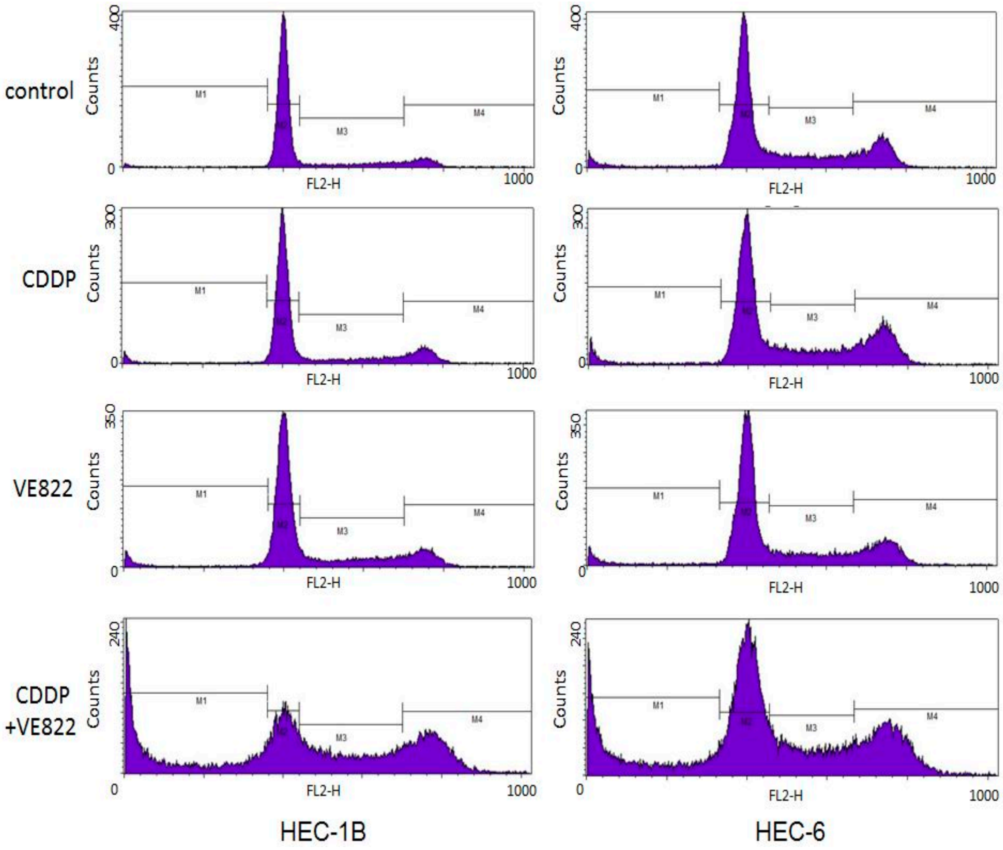

Supplementary Figure S2. Flow cytometric analysis of cell cycle in endometrial cancer cells treated with the combination of DNA-damaging agents and inhibitors. Cells were treated with the drugs for 48 or 72 h and cell cycle distribution was analyzed by flow cytometry. (**a**) DXR (50 nM) and KU60019 (10 μM) incubated for 48 h. (**b**) CDDP (500 nM) and VE822 (100 nM) incubated for 72 h. DXR, doxorubicin; CDDP, cisplatin.

© 2019 by the authors. Licensee MDPI, Basel, Switzerland. This article is an open access article distributed under the terms and conditions of the Creative Commons Attribution (CC BY) license (http://creativecommons.org/licenses/by/4.0/).
